# Multifocal Visual Evoked Potentials (mfVEP) for the Detection of Visual Field Defects in Glaucoma: Systematic Review and Meta-Analysis

**DOI:** 10.3390/jcm10184165

**Published:** 2021-09-15

**Authors:** Haitao Liu, Fei Liao, Román Blanco, Pedro de la Villa

**Affiliations:** 1 Physiology Unit, Department of Systems Biology, School of Medicine, University of Alcalá, 28005 Madrid, Spain; haitao.liu@edu.uah.es (H.L.); fei.liao@edu.uah.es (F.L.); 2 Department of Surgery, School of Medicine, University of Alcalá, 28005 Madrid, Spain; roman.blanco@uah.es; 3 Visual Neurophysiology Group-IRYCIS, 28034 Madrid, Spain

**Keywords:** multifocal visual evoked potentials, mfVEP, glaucoma, perimetry

## Abstract

Some discrepancies have been observed in the diagnostic efficacy of multifocal visual evoked potential (mfVEP) when evaluating visual field defects in glaucoma patients. Therefore, we evaluated the diagnostic precision of the mfVEP in glaucoma to find its best diagnostic indicator. A systematic review and meta-analysis of quantitative studies published up to 1 April 2021 was performed. The methodological quality of the included articles was assessed. Publication bias analysis and heterogeneity tests were performed. The sensitivity, specificity and diagnostic odds ratio were calculated. The area under the curve (AUC) was calculated using the summary of receiver operating characteristics curve. Six studies with a total of 241 patients were included according to the inclusion and exclusion criteria. The AUC was 0.98. There was no evidence of publication bias or threshold effect. The pooled sensitivity and pooled specificity of the mfVEP amplitude for detection of visual field defects in all studies was 0.93 and 0.89, respectively. The positive and negative likelihood ratios of mfVEP amplitude were 6.56 and 0.08, respectively. The amplitude of mfVEP showed a good diagnostic precision in the prediction of visual field defects. Interocular mfVEP amplitude analysis can be a good diagnostic indicator for visual field study.

## 1. Introduction

### Background and Rationale

Glaucoma is the leading cause of irreversible visual damage in the world and incidence is expected to increase to approximately 111.8 million people by 2040 [1]. Therefore, early diagnosis of glaucoma is important to detect and treat patients in the initial stages. Early-stage treatment can prevent its progression to advanced stages and maintain healthy vision.

Glaucoma is an ocular disease characterized by the excavation of the optic nerve and defects in the visual field, constituting one of the main causes of blindness. Intraocular pressure (IOP) can be elevated or normal [2]. Although the mechanism of the pathogenesis of glaucoma is not yet clear, some researchers have proposed different theories about its pathogenesis such as: the intraocular mechanical pressure factor [3,4], the vascular factor [5], anatomical changes in the cribriform plate [6,7], autoimmune factors [8], increase of trans lamina cribrosa pressure [9] and the decrease in intracranial pressure [10].

The standard Humphrey Perimeter 24-2 (SAP) has been used as a standard criterion for measuring visual field defects and diagnosing glaucoma [11]. However, SAP has great variability between test and retest, mainly in areas with decreased visual field sensitivity [12,13]. In addition, SAP is a subjective test that detects visual field abnormalities at a late stage, when up to 25% to 35% retinal ganglion cells (RGC) are lost and statistical abnormalities of 5 dB decrease may be observed in SAP mean deviation [14,15]. Therefore, SAP is not a suitable technique for either the early diagnosis or the monitoring of the evolution of glaucoma. Patient cooperation, physical and psychological factors and the learning effect can also influence the objectivity of the SAP. Finally, the result depends ultimately on the ophthalmologist’s interpretation.

Visual evoked potential (VEP) is a technique that reflects the functional integrity of the visual pathway as a whole. The light response is recorded in the visual cortex, with a flash light stimulus or pattern stimulation on the retina. Deficits in the VEP response do not provide topographical information, so it does not allow local damage to be located [16]. In 1994, Baseler applied multifocal stimuli to record from visual evoked potentials, consisting of 60 black and white checkerboard sectors and performance of VEP [17] with a pseudorandomized m-binary sequence, which allowed the topographical analysis of the response from the retina to the visual cortex. This technique is called multifocal visual evoked potential (mfVEP). It is an objective and sensitive technique to measure the visual field, which does not require manual cooperation when the patient sees the stimulus and the patient only needs to look at the fixation point in the center of the stimulus. It is not affected by physical or psychological factors and it is also observed that age and sex do not affect the objectivity of the patient mfVEP [18].

Multifocal visual evoked potential has been more effective in monitoring unilateral mild damage to ganglion cells than SAP [19,20], while other studies have shown that the amplitude of mfVEP is proportional to the loss in the perimetry of the Humphrey visual field (HVF) 24-2 [11]. The mfVEP has many more stimuli in the central and paracentral region than the traditional HVF 24-2 perimetry, making it more sensitive for detecting central and paracentral defects [21,22]. It has been also shown that mfVEP has higher repeatability reliability than HVF [23]. The mfVEP has also been useful in the diagnosis of diseases such as ischemic and compressive optic neuropathy, optic neuritis and multiple sclerosis [24,25]. Finally, it has been shown that mfVEP amplitude is proportional to glaucoma progression, but just moderately related to mfVEP latency [26,27,28].

To date, there is no standardized protocol for mfVEP that includes stimulation, equipment, electrode placement and the method of analyzing the result. The two types of equipment (Veris and Accumap) are the two main methods included in the literature of mfVEP. In the Veris equipment, the stimulus is a dartboard pattern consisting of 60 sectors. Each sector consists of 8 white checkerboards and 8 black checkerboards and the sizes of the different sectors and checkerboards are scaled according to the cortical magnification of the visual cortex. The patient sits in front of the screen at a viewing angle of 44.5° on the vertical and horizontal axes. Gold cup electrodes are used for recording; one electrode is located 4 cm above the inion, the other two electrodes are placed 4 cm to the left and right side from the point 1 cm above the inion, respectively. The reference electrode is placed on the inion and the ground electrode is placed on the forehead [25]. In the Accumap equipment, the stimulus consists of a cortically scaled dartboard pattern of 58 sectors, with temporal step up to 24° and nasal step up to 32° eccentrically. Each sector consists of four white checkerboards and four black checkerboards. Gold cup electrodes are used for recording; two electrodes are located 4 cm to the left and right side of the inion, one electrode is in the midline 2.5 cm above the inion, one electrode is in the midline for 4.5 cm below the inion and the ground electrode is placed in an ear lobe [29,30]. To interpret the mfVEP results, it has been proposed to calculate the amplitude root mean square (RMS) and the signal-to-noise ratio (SNR) in each sector, then compare each sector with a normative basis to determine its probability, and finally calculate its monocular and interocular cluster [25]. It has been also suggested to calculate the maximum response in each sector and compare it with a normative basis to achieve a probability graph in each sector, then calculate the monocular and interocular scotoma. Calculation of the size, depth and asymmetry of the scotomas gives an index score that is the Accumap Severity Index (ASI) [29,30]. The mfVEP response is similar to VEP, which examines the functional integrity of the visual pathway from the retina to the visual cortex. However, it is not clear which pathway (magnocellular or parvocellular) is stimulated by mfVEP [18,28]. Due to the different ways in which the mfVEP are applied and the results are interpreted, discrepancies have been observed in the diagnostic efficacy of mfVEP latency and amplitude when evaluating visual field defects in glaucoma.

A systematic review seems to be necessary to know whether or not there are significant differences between the distinct ways of applying mfVEP and interpreting its result. It seems also necessary to evaluate the diagnostic accuracy of mfVEP in glaucoma patients and seek its best diagnostic indicator through a systematic diagnostic review of the relevant literature in order to provide useful inspiration for clinical work. The main objective of the present work is to investigate the diagnostic efficacy of mfVEP latency and amplitude to assess visual field defects in glaucoma, and to seek a more precise diagnostic indicator through a systematic diagnostic review of the relevant literature.

## 2. Methods

### 2.1. Search Strategy

The search for articles in this review was performed through the available literature on mfVEP in glaucoma. We searched the online databases PubMed, Medline, Scopus, Embase, Web of Science and the Cochrane Central Register of Controlled Trials, from inception to 1 April 2021.

The search terms were ((“*mfVEP*”) OR (“*multifocal visual evoked potential*”) OR (“*multifocal VEP*”) OR (“*mfVEPs*”)) AND (*glaucoma*) AND ((*human*) OR (*individual*) OR (*patient*) OR (*people*)), later adapted for other databases, and which encompass different terms for mfVEP and glaucoma status. In addition, we examined the reference lists of included studies for potentially eligible works, as well as articles that cited the source to identify any direct/reverse citation.

### 2.2. Inclusion Criteria

Eligibility criteria for this review depended on the PICO framework, which corresponds to Population, Intervention, Comparison and Outcome [31]. Articles that met the inclusion criteria were included. The population included adult patients of both sexes and all ethnicities diagnosed with all types of glaucoma. With at least one eye characterized by glaucomatous optic neuropathy, defined as a cup/disc ratio >0.6, asymmetry of the cup/disc ratio between eyes >0.2, thinning of the rim, notches, excavation and defects of the nerve fiber layer of the retina. A reliable and repeatable visual field defect in SAP, defined as a standard deviation of the pattern <5% and the glaucoma hemifield test outside normal limits. The best corrected visual acuity was at least 20/40, without significant opacities of the ocular media, pupil diameter >3 mm, refractive error not greater than ±6 diopters or two cylinder diopters. No history or presence of other eye and neurological diseases.

This review focused on articles with an observational, cross-sectional and prospective study. Glaucoma patients were confirmed with reference standards such as SAP and optical coherence tomography (OCT), stereo disk photography, Heidelberg retinal tomography (HRT), fundus biomicroscopy or ophthalmoscopy, and then mfVEP with a short interval. Data were compared with a control group including healthy people without eye diseases. The reviewed results included diagnostic indicators such as mfVEP amplitude or mfVEP latency and then assay methods such as cluster or ASI, sensitivity, specificity, true positive (TP), false positive (FP), true negative (TN), false negative (FN) were estimated. TP, FN, FP, TN are defined as: in the glaucoma patient group diagnosed with the reference standard, the mfVEP analysis result is positive (TP) or negative (FN); in the control group with healthy people confirmed by the reference standard, and the mfVEP analysis result is positive (FP) or negative (TN).

### 2.3. Exclusion Criteria

Studies with any of the following characteristics were excluded: (a) No control group of healthy people; (b) not on human beings; (c) systematic reviews; (d) incomplete data on evaluation index; (e) reports of cases with less than 10 people.

### 2.4. Selection of Studies

Data selection and extraction were performed by two independent investigators based on eligibility characteristics such as population, intervention, comparison and study types. We first reviewed the titles and abstracts. If the articles matched our inclusion criteria, we read the full texts to make a final decision for the inclusion of each study. If there was any discrepancy, we requested the opinion of a third investigator. Finally, we made a selection flow diagram, the preferred items report for systematic reviews and meta-analysis (PRISMA) for the study [32].

The management of the articles was carried out in the Endnote software (Endnote X8, Clarivate Analytics, London, UK). First, we excluded duplicate items. By analyzing titles, abstracts and keywords, we eliminated articles that were not relevant to the study of mfVEP in glaucoma. We also checked the reference list of review articles so as not to miss a primary research study. Finally, we read the full text. Articles that met the strict inclusion criteria underwent data extraction.

### 2.5. Data Extraction

The data were extracted in a form that included the following variables:

- Basic information on the study such as country of origin, year of publication and sample size;
- Information on the study design, such as an observational, cross-sectional, prospective study;
- Participant information, such as age, gender, ethnicity and severity of glaucoma;
- Experimental information such as reference standard, equipment, diagnostic indicator, amplitude or latency of the mfVEP and the assay method such as cluster or ASI analysis;
- Key results such as sensitivity and specificity, TP, FP, TN, FN.

We did not have the approval of any ethical research committee, since our study was a secondary analysis of the publications, which did not directly involve any human subject.

### 2.6. Assessment of Risk of Bias

The assessment of the methodological quality of the included studies was carried out using the tool for assessing the Quality of Diagnostic precision Studies, version 2 (QUADAS-2) [33]. A total of 14 assessment items were established and each study was scored according to ‘yes’, ‘no’ and ‘unclear’. In QUADAS-2, there are four key domains to evaluate the risk of bias: (1) the selection of patients, (2) the index test, (3) the reference standard and (4) the time between the index test and the reference standard and the flow of the patient in the study.

The first three domains above also evaluate applicability with respect to participants, equipment, performance and interpretation. Furthermore, the diagnosis of disease according to the reference standard coincides or does not coincide with our review [34]. The risk of bias section assesses the design of the included studies and their potential for bias. The applicability concerns section assesses the relationship between the included studies and our review question and whether or not the included studies matched our review question. Two investigators evaluated each article independently. If they did not reach a consensus, it was resolved with the judgment of another researcher.

### 2.7. Data Synthesis and Statistical Analysis

#### 2.7.1. Heterogeneity Test

We used the chi-square (X^2^) and Cochran-Q statistical tests to assess heterogeneity between the included studies; a result with a low *p* value indicated heterogeneity. The inconsistency index (I^2^) was also calculated to quantify heterogeneity; an I^2^ value of 0% means there is no heterogeneity and an I^2^ value greater than 50% means there is heterogeneity between the included studies [35].

#### 2.7.2. Threshold Effect

In a study for the assessment of test precision, the threshold effect may be a major cause of heterogeneity due to the lack of standardization of the definition of a positive result. To find if there is a threshold effect, we can calculate the Spearman correlation coefficient between the sensitivity logit and the 1-specificity logit; a positive correlation and a *p* < 0.05 indicate the existence of a threshold effect. Furthermore, the presence of a typical “shoulder-arm” pattern on the receiver operating characteristic (ROC) curve signifies a possible threshold effect [36,37].

#### 2.7.3. Publication Bias

Studies with favorable and optimistic results are more likely to be accepted and published; this may be a factor that influences the conclusions [38]. We evaluated the publication biases through the funnel plot. The graph with symmetrically distributed data points means the absence of publication bias and an asymmetric graph indicates the existence of publication bias [39].

### 2.8. Statistical Summary

The extracted data were used to construct forest plots of sensitivity and specificity and to estimate the diagnostic accuracy of mfVEP in the diagnosis of visual field defects in glaucoma patients. The statistical summary can be calculated using the fixed effects model (FEM) or using the random effects model (REM) depending on the homogeneous characteristics of the included studies. We used the bivariate regression method to calculate sensitivity and specificity. We then summarized the corresponding positive likelihood ratio (PLR), negative likelihood ratio (NLR) and diagnostic odds ratio (DOR). Summary receiver operating characteristic curve (sROC) synthesis is based on pooled sensitivity, pooled specificity and respective variations [40,41]. The area under the curve (AUC) of sROC is used to evaluate the overall performance of the test [42]. DOR is calculated by combining sensitivity and specificity [43].

### 2.9. Sensitivity Analysis

We performed a sensitivity analysis by sequential elimination of the studies, in order to avoid those studies that would affect results in a statistically significant way. When there was significant heterogeneity, the source of the heterogeneity was sought and, when necessary, a subgroup analysis was performed between the included studies. Subgroup analysis was appropriate among participants of varying glaucoma severity, ethnicity, equipment, mfVEP assay method and number of populations.

The analyses of heterogeneity, sensitivity, threshold effect and calculation of the statistical estimate were performed with Meta-Disc version 1.4 (Hospital Ramón y Cajal. Madrid, Spain) [37]. The publication bias analysis was performed using Stata 15.0 (StataCorp LP, College Station, TX, USA).

## 3. Results

### 3.1. Selection of Studies

**Figure 1:**
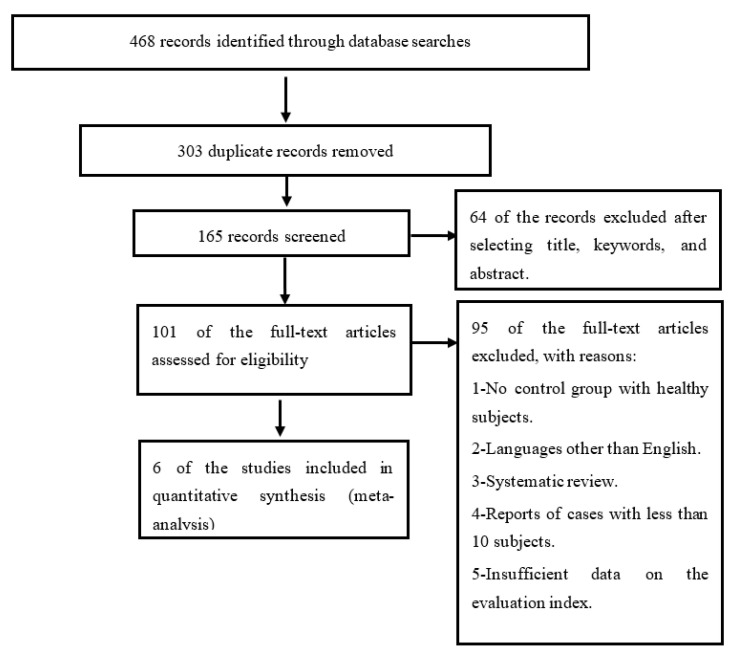
Flowchart of studies included through the systematic review process.

Figure 1 shows the complete flow of the selection of the studies included in the present work. Initially, 468 total articles were found by the search carried out through the different databases. Of the total articles, 303 were duplicates and 159 articles were excluded because they were not considered relevant for the systematic review proposed. The reasons for exclusion were: (i) the article did not include a control group with healthy subjects; (ii) articles written in a language other than English; (iii) systematic reviews; (iv) articles and reports of cases with a small number of participants; (v) the article provided insufficient data on the evaluation index (true positives, false positives, true negatives and false negatives). Finally, a total of six studies were included [20,44,45,46,47,48] which met all the inclusion criteria.

### 3.2. Characteristics of the Included Studies

In the six included studies, a total of 241 patients (273 eyes) diagnosed with glaucoma and 195 healthy people (202 eyes) could be counted. Of the 241 patients, 196 belonged to the mixed-type glaucoma group (different types of glaucoma), while 10 patients had normal tension glaucoma (NTG) and the other 35 patients had primary open-angle glaucoma (POAG). Table 1 shows the main characteristics of the six studies included in the meta-analysis.

### 3.3. Methodological Quality of the Included Studies

**Figure 2:**
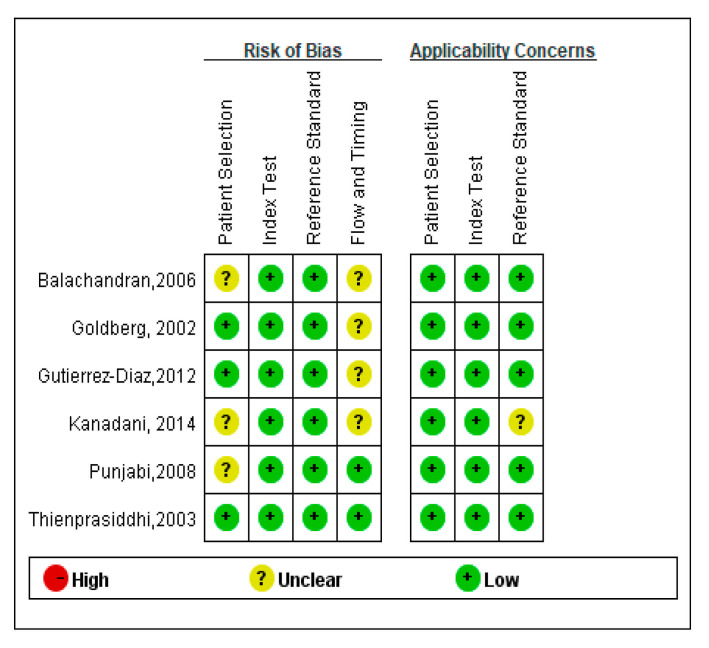
Quality of the included studies according to the tool for assessing the quality of diagnostic precision studies (QUADAS). The results revealed that the six studies included in this review met 9 or more of the 14 criteria of the QUADAS tool, indicating relatively good methodological quality of the included studies.

The methodological quality of the six reviewed studies was initially assessed with the QUADAS-2 tool. Four of the six trials were cross-sectional studies; three trials enrolled patients consecutively and other enrollments were not specified. Gold standard tests included SAP to detect visual field loss, OCT, stereo disc photography, HRT, fundus biomicroscopy, or ophthalmoscopy to study the findings of the glaucomatous optic nerve. Only one of the articles mentioned the time interval between the mfVEP index test and the reference standard; the others did not mention the interval time, indicating a high risk of bias in the flow and time domain. The result of the evaluation of the methodological quality of the included studies is shown in Figure 2.

### 3.4. Synthesis of Diagnostic Data

**Figure 3:**
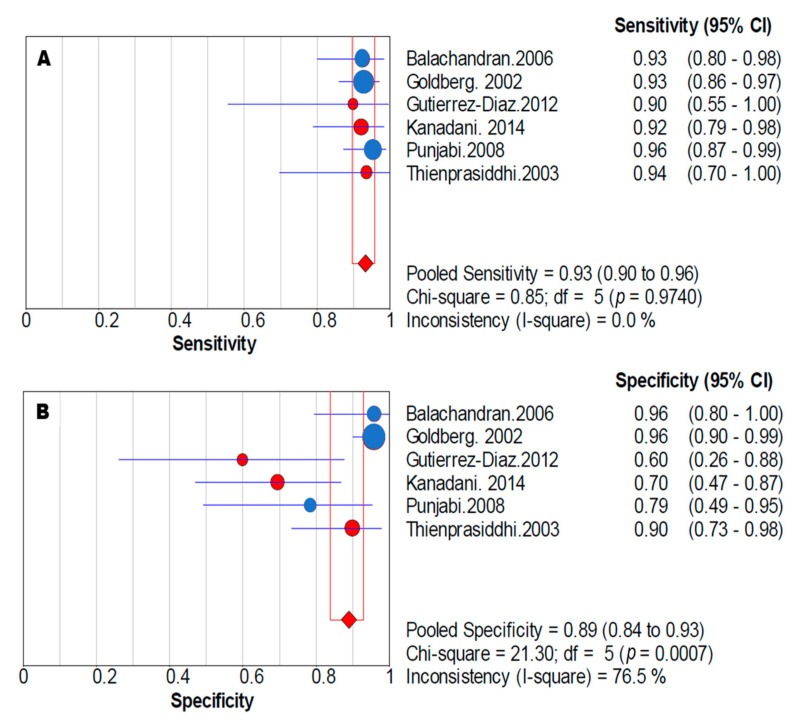
Sensitivity and specificity forest plots in the meta-analysis. Forest plots of sensitivity (**A**) and specificity (**B**) corresponding to the amplitude of mfVEP to diagnose visual field defects in glaucoma. Pooled sensitivity was 0.93 (95% CI: 0.90–0.96) and pooled specificity was 0.89 (95% CI: 0.84–0.93). The size of each point is proportional to the study sample size or the weight of the study in the meta-analysis. The blue points represent studies with Accumap equipment and the red points are with Veris equipment.

The summary estimates of the mfVEP amplitude for the diagnosis of visual field defects in the six studies corresponded to a sensitivity of 0.93 (95% confidence interval, CI: 0.90–0.96) and a specificity of 0.89 (95% CI: 0.84–0.93). The positive likelihood ratio was 6.56 (95% CI: 2.67–16.10), the negative likelihood ratio was 0.08 (95% CI: 0.05–0.12) and the diagnostic odds ratio was 90.00 (95% CI: 31.51–257.11) (Table 2). Figure 3 shows the forest plots of sensitivity and specificity.

### 3.5. Heterogeneity and Threshold Effect

In our meta-analysis, no signs of heterogeneity were observed for sensitivity results (I^2^ = 0%), but substantial heterogeneity was observed for specificity (I^2^ = 73.4%).

To find the source of heterogeneity, we performed a subgroup analysis based on the mfVEP registry team (Veris and Accumap), assay methods (ASI and Cluster) and glaucoma severity depending on SAP mean deviation (MD). However, heterogeneity for specificity remained high, with an inconsistency rate (I^2^) greater than 50%, while heterogeneity for sensitivity in all subgroups was I^2^ = 0%. Our review includes just 241 glaucoma patients and six articles in the meta-analysis, and we are aware that when a meta-analysis includes fewer than 500 patients and fewer than 15 trials, there may be fluctuations in I^2^ estimates [49].

After excluding articles one by one through sensitivity analysis, the inconsistency rate for sensitivity heterogeneity was found to be 0%. However, the inconsistency rate for specificity was greater than 50%. Sensitivity, specificity, area under the curve (AUC) and 95% confidence interval (CI) were similar and overlapped with each other. The heterogeneity remained the same. Sensitivity analysis showed that the pooled estimates were stable and reliable.

A Spearman rank correlation was performed, which provided a value of −0.464 (*p* = 0.354); in addition to the sROC, a “shoulder-arm” pattern was not appreciated, which means that there was no threshold effect between the included studies.

**Figure 4:**
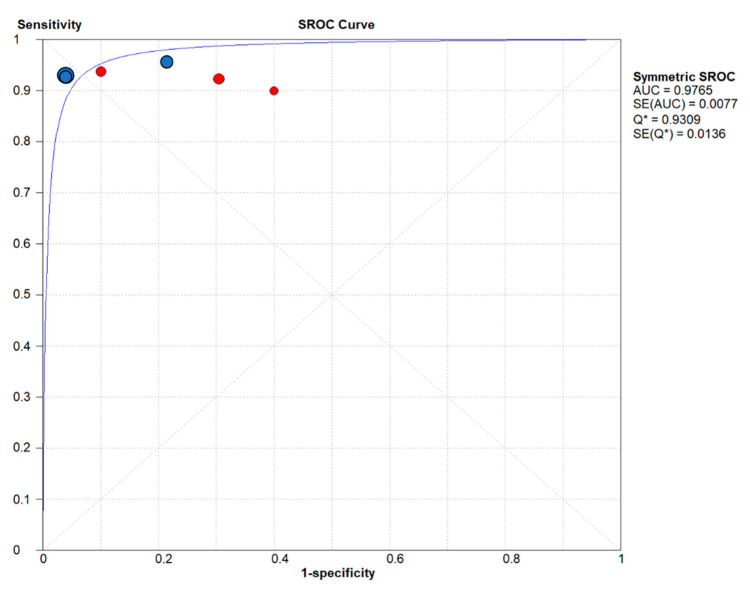
Summary receiver operating characteristic (sROC) curve for the six included studies. The area under the ROC summary curve was observed to be approximately 1.0. The sROC synthesis was based on pooled sensitivity, pooled specificity and respective variances. The size of each point is proportional to the study sample size or the weight of the study in the meta-analysis. The blue points represent studies with Accumap equipment and the red points are with Veris equipment. AUC = Area Under the Curve. Q = Index Q. SE = Standard Error.

We calculated the AUC of the sROC to evaluate the overall performance of the mfVEP amplitude for the diagnosis of visual field defects in glaucoma (Figure 4) and the value was 0.97. Due to the heterogeneity that existed in specificity, we did the statistical summary using the random effects model.

A funnel plot was also constructed to assess publication bias in the meta-analysis. The data points had a symmetric funnel shape, meaning the absence of publication bias. However, we must point out that in our study we only included six articles, which is a relatively small study group to do a publication bias analysis, since it is recommended that there be a minimum of 10 studies [50,51].

From the obtained results, we can confirm that the quality of the six included studies was relatively good. Diagnostic data, including sensitivity, specificity, likelihood ratio and area under the sROC curve, demonstrated that mfVEP amplitude had good diagnostic accuracy in predicting visual field defects in glaucoma patients and in the analysis of the interocular mfVEP amplitude. Therefore, it can be a good diagnostic indicator for the visual field study.

## 4. Discussion

In this systematic review, the diagnostic efficacy of mfVEP in evaluating visual field defects in glaucoma patients was reviewed, using the amplitude and latency of its responses as diagnostic indicators. Regarding the amplitude, the results show a pooled sensitivity of 0.93 (95% CI: 0.90–0.96) and a pooled specificity of 0.89 (95% CI: 0.84–0.93). The positive likelihood ratio was 6.56 (95% CI: 2.67–16.10), the negative likelihood ratio was 0.08 (95% CI: 0.05–0.12) and the diagnostic odds ratio was 90.00 (95% CI: 31.51–257.11). The area under the curve of the summary receiver operating characteristic was 0.97, indicating a good performance of the amplitude recorded by the mfVEP in the prediction of visual field defects in glaucoma. The results of the sensitivity analysis demonstrated that the pooled estimates are stable and reliable.

In our meta-analysis, the heterogeneity for pooled specificity was somewhat high (I^2^ = 73.4%) and no heterogeneity was observed for pooled sensitivity (I^2^ = 0%). However, it is important to note that our study does not include a high number of patients, which may have been the cause of fluctuations in the estimate of I^2^ [49]. The existence of heterogeneity in the pooled specificity indicates that the low number of people in the control group may be the cause of variation in specificity between the different studies. In our study, both pooled specificity and heterogeneity are high, in contrast to those observed in some studies when very low specificity was found [21,52]. We also found a wide variety of specificity between different studies [52]. In fact, it was suggested that the low number of people evaluated may be a possible cause of the variability observed in the specificity. Another possible cause is the lack of operator training or the erroneous or too narrow normal limits in the amplitudes recorded by the mfVEP [52].

The analysis of the subgroups evaluated with different equipment (Veris and Accumap) and test methods (ASI and Cluster) are the two main methods included in the literature on mfVEP, which show certain differences such as the number of stimulus sectors, the placement of the electrodes or how to interpret the results [25,29,30]. However, no significant differences were observed in their visual field diagnostic accuracy.

Regarding the latency of mfVEP in the diagnosis of visual field defects, some articles found a low correlation with the result of SAP [26,27,28]. In our review, we did not find enough articles with diagnostic data to perform a meta-analysis of the latency recorded by mfVEP.

In all the studies included in our review, both mono and interocular amplitude analysis are included. However, according to the data obtained in the study, diagnostic precision could not be assessed with the monocular amplitude analysis alone. The results showed significant variability among the patients, mainly due to the existing anatomical differences in the visual cortex and the position of the calcarine cortex in relation to the location of the external electrodes, as well as differences in the cortical folds [53]. The interocular analysis of the same individual makes it possible to reduce the variability between participants. In fact, this can be a disadvantage of mfVEP compared to SAP. On the other hand, mfVEP is more sensitive in patients with glaucoma and asymmetric visual field loss [19,54]. Furthermore, the mfVEP has more stimulus sectors at a center of 10 degrees. Therefore, it is more sensitive to the detection of central damage [55]. However, the detection of damage in the superior and peripheral campimetries is more complex, since it corresponds to the deep cortical area, located behind the calcarine fissure.

Our systematic review has confirmed that mfVEP amplitude may be a diagnostic or prognostic biomarker of visual defects in glaucoma. Doubtful clinical cases due to unconfirmed visual field defects based on dissociation between OCT-SAP, cases of unreliable SAP, concentration or motor problem of the patient to perform SAP, should use mfVEP amplitude as a complementary, reliable and objective tool. In this sense, the mfVEP technique is non-invasive, does not require subjective patient cooperation and each test is inexpensive. Its main disadvantage is that its application requires a well-trained expert.

We cannot discard some limitations in our systematic review. The meta-analysis only included 241 patients and six articles, which in our opinion is few cases if we want to evaluate the diagnostic efficacy of mfVEP in predicting visual field defects in glaucoma. Moreover, several studies used different techniques to study glaucomatous findings of the optic nerve, such as OCT, optic disc photography, HRT, fundus biomicroscopy or ophthalmoscopy. Just one article mentions the time interval between the index test and the reference standard, while the others do not indicate the time of the interval, which means a high risk of bias in the domain flow and timing. Furthermore, several articles did not mention whether they interpreted the results of the index test and the reference standard without knowing the results of the others, which is a factor that can cause inflamed measures in the diagnostic test. Finally, there are also different mfVEP parameters, such as being divided into four quadrants, six sectors, several rings, percentage of abnormal points, hemifields, etc., but we could not analyze them because few publications met our criteria.

## 5. Conclusions

The amplitude of mfVEP has shown good diagnostic accuracy in predicting visual field defects in glaucoma patients. Interocular mfVEP amplitude analysis can be a diagnostic indicator for visual field study in doubtful or unreliable cases of automated standard perimetry.

## Figures and Tables

**Table 1:** Main characteristics of the studies included in the meta-analysis.

ID Study	Type of Study	Types and No. of Glaucoma Patients	Glaucoma Severity (MD of SAP) dB	Reference Standard	Equipment for Index Test (mfVEP)	Diagnostic Indicator-Assay Method	Age (Years) Mean ± SD
Balachandran, 2006	NA	Mixed 41P 41E	−7.1 ± 6.0	SAP, stereoscopic optic nerve head photography, HRT	AccuMap version 2.0	Amplitude- ASI	65 ± 11
Goldberg, 2002	Cross	Mixed 100P 100E	−6.5 ± 4.2	SAP, stereo disk photography	AccuMap version NA	Amplitude- ASI	62.2 ± 9.8
Gutierrez-Diaz, 2012	Cross	NTG 10P 10E	−5.95 ± 11.7	SAP, OCT, ophthalmoscopy	VERIS version NA	Amplitude- Cluster	66.8 ± 6.1
Kanadani, 2014	Cross	Mixed 39P 39E	all levels	SAP, fundus biomicroscopy	VERIS version NA	Amplitude- Cluster	66.3 ± NA
Punjabi, 2008	Cross	POAG 35P 67E	−6.2 ± 0.8	SAP, indirect ophthalmoscopy, HRT	AccuMap version 2.0	Amplitude- ASI	68 ± 10
Thienprasiddhi, 2003	NA	Mixed 16P 16E	−6.8 ± 4.2	SAP, stereoscopic optic nerve head photography	VERIS version 4.3	Amplitude- Cluster	56 ± 7

NA = Not available. Cross = Cross-sectional study. P = People. E = Eyes. POAG = Primary open angle glaucoma. NTG = Normal tension glaucoma. Mixed = Different types of mixed glaucoma. SAP = Standard automated perimetry. MD = Mean deviation. OCT = Optical coherence tomography. HRT = Heidelberg Retinal Tomography. BMC = Biomicroscopy. ASI = Accumap Severity Index. The study identification (ID) numbers correspond to the study numbers in the graphs of Figure 2 and Figure 3.

**Table 2:** Statistical summary of the diagnostic precision parameters in the meta-analysis.

Parameter	Estimates
Total eyes (*n*)	475
True positive (*n*)	255
False negative (*n*)	18
False positive (*n*)	22
True negative (*n*)	180
Accuracy (*n*)	0.92 (95% CI: 0.89 to 0.94)
Sensitivity	0.93 (95% CI: 0.90 to 0.96)
Specificity	0.89 (95% CI: 0.84 to 0.93)
PLR	6.56 (95% CI: 2.67 to 16.10)
NLR	0.08 (95% CI: 0.05 to 0.12)
DOR	90.00 (95% CI: 31.51 to 257.11)

Statistical summary of diagnostic accuracy parameters for mfVEP amplitude in diagnosing visual field defects in glaucoma patients included accuracy, pooled sensitivity, pooled specificity, positive likelihood ratio (PLR), negative likelihood ratio (NLR), the diagnostic odds ratio (DOR) and their 95% confidence interval (CI), respectively.

## Data Availability

Not applicable.
